# *Nuclear Protein 1* Expression Is Associated with PPARG in Bladder Transitional Cell Carcinoma

**DOI:** 10.1155/2023/6797694

**Published:** 2023-05-08

**Authors:** Chao Lu, Shenglin Gao, Li Zhang, Xiaokai Shi, Yin Chen, Shuzhang Wei, Li Zuo, Lifeng Zhang

**Affiliations:** Department of Urology, Changzhou Second People's Hospital, 29 Xinglong Road, Changzhou 213003, China

## Abstract

**Background:**

The *Nuclear protein 1* gene was first discovered in acute pancreatitis and functions as an oncogene in cancer progression and drug resistance. However, the role of *Nuclear protein 1* in bladder transitional cell carcinoma (BTCC) is still unclear.

**Methods:**

The Cancer Genome Atlas database and immunohistochemical analysis were adopted to evaluate *Nuclear protein 1* expression in BTCC. We applied lentivirus-mediated small-interfering RNA to down-regulate the expression of *Nuclear protein 1* in BTCC cell lines. We further performed an Affymetrix microarray and Gene Set Enrichment Analysis (GSEA) to assess the genes and signaling pathways related to *Nuclear protein 1*.

**Results:**

We found that *Nuclear protein 1* expression was up-regulated in BTCC and positively related to the degree of BTCC malignancy. Compared with Caucasian patients with BTCC, *Nuclear protein 1* expression was attenuated in Asian patients. The Affymetrix microarray showed that lipopolysaccharide was the upstream regulatory factor of *Nuclear protein 1* in BTCC. The GSEA indicated that *Nuclear protein 1* expression was associated with signaling pathways in cancer, peroxisome proliferator-activated receptor (PPAR) pathways, and RNA degradation. The expression of *Nuclear protein 1* was negatively correlated with PPARG (*R* = −0.290, *P* < 0.001), but not with PPARA (*R* = 0.047, *P* = 0.344) and PPARD (*R* = −0.055, *P* = 0.260).

**Conclusions:**

The study findings indicate that *Nuclear protein 1* is positively associated with the malignancy degree of BTCC and that *Nuclear protein 1* expression is negatively correlated with PPARG.

## 1. Introduction

Bladder transitional cell carcinoma (BTCC) is one of the most common malignancies of the urinary system. In the United States, 61,700 new cases of BTCC in men and 19,480 new cases in women were estimated in 2022. A total of 12,120 men and 4,980 women died in the same year as a result of BTCC [1]. BTCC is also one of the five most common malignancies in the United States. Given its high recurrence rate, BTCC remains one of the most expensive malignancies to treat [2]. With about 550,000 new patients each year, BTCC is one of the 10 most common malignancies worldwide. Currently, developed communities have the heaviest burden of BTCC [3], and hence, strategies are required for the prevention and control of BTCC and to alleviate the exorbitant burden on the society and economy. The gradual understanding of urinary biomarkers in BTCC has facilitated the employment of non-invasive biomarkers to replenish urine cytology. However, none is effective enough when performed alone, and pathology remains to be the gold standard method to detect and diagnose BTCC [4].

The *Nuclear protein 1* (also named Com-1) is a gene strongly up-regulated in the acute stage of pancreatitis. The *Nuclear protein 1* mRNA is activated in response to a variety of stress responses, and its activation is not limited to pancreatic cells. Restoration of *Nuclear protein 1* expression in transformed fibroblasts results in the formation of carcinoma, suggesting that the expression of *Nuclear protein 1* is essential for tumor development [5, 6]. The *Nuclear protein 1* is a protein associated with a high-migration subgroup of transcriptional regulatory proteins and plays a hinge role in cellular stress response and metastasis [7]. The CAAT-enhancer binding protein (C/EBP) is a cis-acting element at the nucleotide −111 position of *Nuclear protein 1* and can facilitate the transcription of the *Nuclear protein 1* gene in mice [8]. The anomalous expression of *Nuclear protein 1* in many benign disorders can cause renal mesangial cell hypertrophy, cardiac fibrosis, and higher autophagy [9–11]. Additionally, as a transcription regulator, *Nuclear protein 1* can participate in DNA damage response, cell cycle, apoptosis, and chromatin remodeling in response to chemotherapeutic resistance in carcinoma [12]. Understanding the regulation of multifaceted functions of *Nuclear protein 1* can provide new insights, which could help in the creation of new therapies for cancer and other pathologies [13]. Moreover, conclusions from existing *in vitro* studies may not be in line with those from *in vivo* studies [14].

Initially, *Nuclear protein 1* was thought to regulate pancreatic cancer cell growth through growth suppression-related pathways and the inhibition of cell growth promoting factors [15]. To a certain extent, *Nuclear protein 1* adjusts the migration, invasion, and adhesion of pancreatic cancer cells through cytoskeletal regulatory factors [16]. Results from breast cancer studies revealed that *Nuclear protein 1* interacts with p53 to up-regulate the anti-apoptotic protein Bcl-2, giving breast epithelial cells an advantage in growth and survival [17]. As a transcriptional co-regulator, *Nuclear protein 1* plays a key role in the endocrine therapy of breast cancer, thus representing a sensitive therapeutic target for the study of endocrine resistance of breast cancer [18]. *In vitro* studies revealed *Nuclear protein 1* to be a potential tumor suppressor in human prostate cancer, and that *Nuclear protein 1* expression is inversely associated with prostate cancer aggressiveness and growth [19]. The specific mechanism of *Nuclear protein 1* action in BTCC cells and tissues remains unclear till date. Therefore, the present study assessed the expression of *Nuclear protein 1* in clinical BTCC tissues. In addition, the regulatory factors of *Nuclear protein 1* and the signaling pathway of their interaction were identified through functional experiments. Bioinformatic tools were used to verify *Nuclear protein 1*-related genes and signaling pathways in BTCC.

## 2. Materials and Methods

### 2.1. Study Population

RNA sequencing data and corresponding clinicopathological data of 414 cases of BTCC were extracted from The Cancer Genome Atlas (TCGA) database. The baseline data sheet of patients with BTCC who were enrolled for the study is summarized in Supplemental Table 1. The specimens collected from these patients were first examined by pathological examination. Patients who had received chemotherapy or radiation before operation were excluded. In addition, we recruited patients with BTCC from our research center and conducted clinical verification on the tissue samples of these patients. Changzhou Second People's Hospital's Ethics Committee approved the study protocol.

### 2.2. Immunohistochemical Analysis

Immunohistochemical analysis was employed to verify the clinical samples of patients with BTCC enrolled in our center. The control groups were derived from cancer-free mucosal tissue from patients with BTCC. The slices were stained with anti-*Nuclear protein 1* antibody using standard immunoperoxidase-staining protocols. Two pathologists were invited to evaluate the tissue sections and obtain the corresponding staining scores. We independently chose five fields of view for each section. The staining intensity score was recorded as four scales (0–3) according to the number of immune response cells.

### 2.3. Cell Culture

We used the human BTCC cell line (5637 Cell Line, Shanghai, China) for functional experiments. This cell line was cultured in a RPMI-1640 medium (Gibco, USA). The medium consisted of 10% fetal bovine serum as well as 100 U/mL penicillin and streptomycin. The cells were stored in a humidified incubator with 5% CO_2_ at 37°C.

### 2.4. Transfection of Lentivirus and Affymetrix Microarray Analysis

To construct lentivirus with low *Nuclear protein 1* expression, we designed the RNA interference sequence (RNAi) system using a part of *Nuclear protein 1* sequence (CCAAGCTGCAGAATTCAGA). A non-silencing small-interfering RNA (siRNA) sequence (TTCTCCGAACGTGTCACGT) was used as negative control. We employed the PrimeView Human Gene Expression Array (Affymetrix, Thermo Fisher Scientific, USA) and conducted gene chip assays to explore the differential expression profile after *Nuclear protein 1* interference. Total RNA was retrieved from *Nuclear protein 1* interference and control cells. The NanoDrop 2000 (NanoDrop Technologies, Wilmington, USA) was used to evaluate the quality of the total RNA, and the GeneChip kit (Affymetrix, Thermo Fisher Scientific, USA) was used to perform gene hybridization, washing, and staining based on the manufacturer's instructions. Subsequently, an ingenuity pathway analysis (IPA) was carried out to annotate the gene microarray expression profiles.

### 2.5. Western Blotting

After quantification, the total protein (<100 *μ*g) was mixed with protein marker and buffer. The buffer was subsequently added into the wells of a sodium dodecyl sulfate–polyacrylamide gel electrophoresis gel for electrophoretic separation. Afterwards, we transferred the samples to a polyvinylidene fluoride membrane, which was further blocked with 5% skim milk. The *Nuclear protein 1* antibody was purchased from Proteintech Group. The BCL-2, BAX, E-cadherin, vimentin, P21, N-cadherin, cyclin-D1, C-caspase 3, CDK2, and GAPDH antibodies were bought from Abcam company. GAPDH was used as the internal control. The membranes were then incubated with an appropriate primary antibody overnight at 4°C. After a thorough wash, the samples were reacted with a secondary antibody for 2 hours (20°C). We used the chemiluminescence reagent (Millipore, USA) to evaluate the protein bands.

### 2.6. GSEA and Bioinformatic Analyses of *Nuclear Protein 1*

We used the Gene Set Enrichment Analysis (GSEA) to assess the possible pathways associated with *Nuclear protein 1*. A gene set, c2.cp.kegg.v7.1.symbols.gmt, was chosen as the reference gene set [20]. We adopted the R language to analyze the clinical data acquired from the TCGA database and applied the Search Tool for the Retrieval of Interaction Gene/Proteins (STRING) server to explore the protein–protein crosstalk of *Nuclear protein 1* in Homo sapiens (https://string-db.org/cgi/input.pl). We then used the GraphPad Prism software to evaluate the findings of immunohistochemical analyses. Participants were classified into two groups according to the *Nuclear protein 1* expression. We employed a prognostic classifier to explore whether the expression of *Nuclear protein 1* influences the clinical outcomes in patients with BTCC. The rank of differentially expressed genes associated with *Nuclear protein 1* was measured by R (3.6.3). We applied the predictive receiver operating characteristic package to create receiver operating characteristic (ROC) curves, and multivariate Cox analysis was used to evaluate the influence of *Nuclear protein 1* expression on prognosis. We employed the University of ALabama at Birmingham CANcer data analysis Portal (http://ualcan.path.uab.edu/analysis.html) to explore the expression profile of PPARG in bladder cancer. The influence of different PPARG expression levels on overall survival and disease-free survival time was investigated by Gene Expression Profiling Interactive Analysis (GEPIA, http://gepia.cancer-pku.cn/index.html) database. We further used a correlation chord diagram to outline the degree of correlation between *Nuclear protein 1*, PPARG, PPARA, and PPARD. The nomogram chart was based on multivariate regression analysis. Statistical significance was set at *P* < 0.05.

## 3. Results

### 3.1. Expression of *Nuclear Protein 1* in Clinical Tissue of BTCC

**Figure 1:**
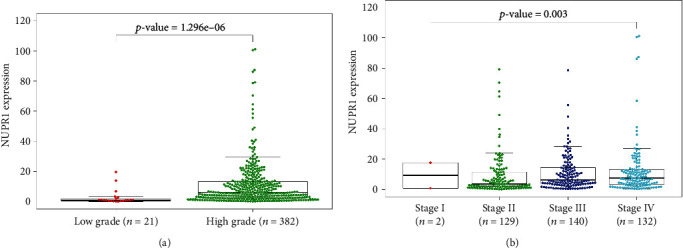
Association between *Nuclear protein 1* expression and clinical manifestation of bladder transitional cell carcinoma (BTCC) evaluated by TCGA database. (a) The *Nuclear protein 1* in high-grade BTCC was enhanced in low-grade BTCC (*P* < 0.05). (b) The *Nuclear protein 1* was positively associated with the stage of BTCC (*P* < 0.05).

**Figure 2:**
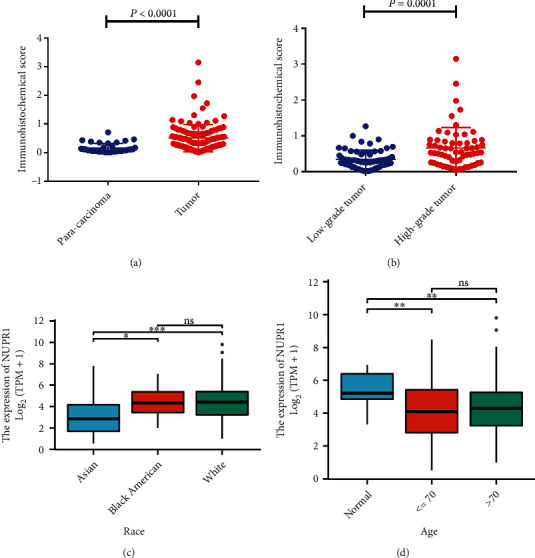
Expression of *Nuclear protein 1* in different subgroups of BTCC. (a) The expression of *Nuclear protein 1* was down-regulated in para-carcinoma tissues (*P* < 0.001). (b) The *Nuclear protein 1* in high-grade BTCC was significantly augmented than that in low-grade BTCC (*P* < 0.001). (c) Compared with Caucasian patients with BTCC, the *Nuclear protein 1* expression was attenuated in Asian patients. (d) There was no difference in the expression of *Nuclear protein 1* in patients with different ages.

The TCGA database was employed to demonstrate the association between *Nuclear protein 1* and clinical traits of BTCC. The expression of *Nuclear protein 1* in high-grade BTCC was higher than that in low-grade BTCC (Figure 1(a), *P* < 0.05). Moreover, *Nuclear protein 1* expression was positively associated with the clinical stage of BTCC (Figure 1(b), *P* < 0.05). Further, the tissues collected from patients with BTCC were subjected to immunohistochemical analysis of *Nuclear protein 1*. Our results provide evidence that *Nuclear protein 1* is down-regulated in para-carcinoma tissues (Figure 2(a), *P* < 0.001). Furthermore, *Nuclear protein 1* expression in high-grade BTCC was significantly augmented compared to that in low-grade BTCC (Figure 2(b), *P* < 0.001).

### 3.2. *Nuclear Protein 1* Expression in Different Subgroups of BTCC

**Figure 3:**
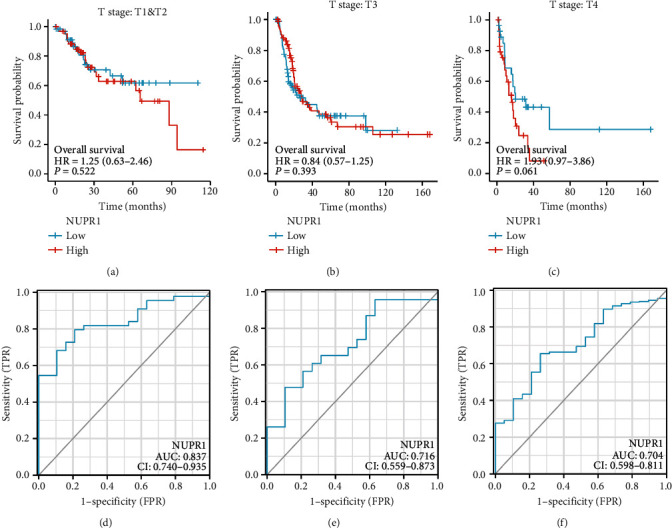
Prognosis analysis of *Nuclear protein 1* in different stage of BTCC. High *Nuclear protein 1* expression had a worse prognosis in patients with advanced BTCC (c) compared to relatively early cancer (a) and (b). ROC curve of *Nuclear protein 1* expression in Asian, Caucasian, and Black American subgroup was revealed in (d), (e), and (f).

We further utilized the R language to explore the expression of *Nuclear protein 1* in different subgroups of BTCC. Details of the *Nuclear protein 1* expression in different ethnic groups were summarized in Supplemental Table 1. The logistic regression model was used to analyze the odds ratio (OR) in different subgroups of BTCC (Supplemental Table 2). Compared with Caucasian patients with BTCC, *Nuclear protein 1* expression was attenuated in Asian patients (Figure 2(c), *P* < 0.001). No difference was observed in the expression of *Nuclear protein 1* among patients with different ages (Figure 2(d), *P* > 0.05). Additionally, we analyzed the prognosis potential of *Nuclear protein 1* in different stages of BTCC. High *Nuclear protein 1* expression had a worse prognosis in patients with advanced BTCC (Figure 3(c)) compared with patients with relatively early cancer (Figures 3(a) and 3(b)). The ROC curves of *Nuclear protein 1* expression in Asian, Caucasian, and Black American subgroups are shown in Figures 3(d), 3(e), and 3(f).

### 3.3. Investigation of the Relative Regulatory Molecules of *Nuclear Protein 1* via Microarray

**Figure 4:**
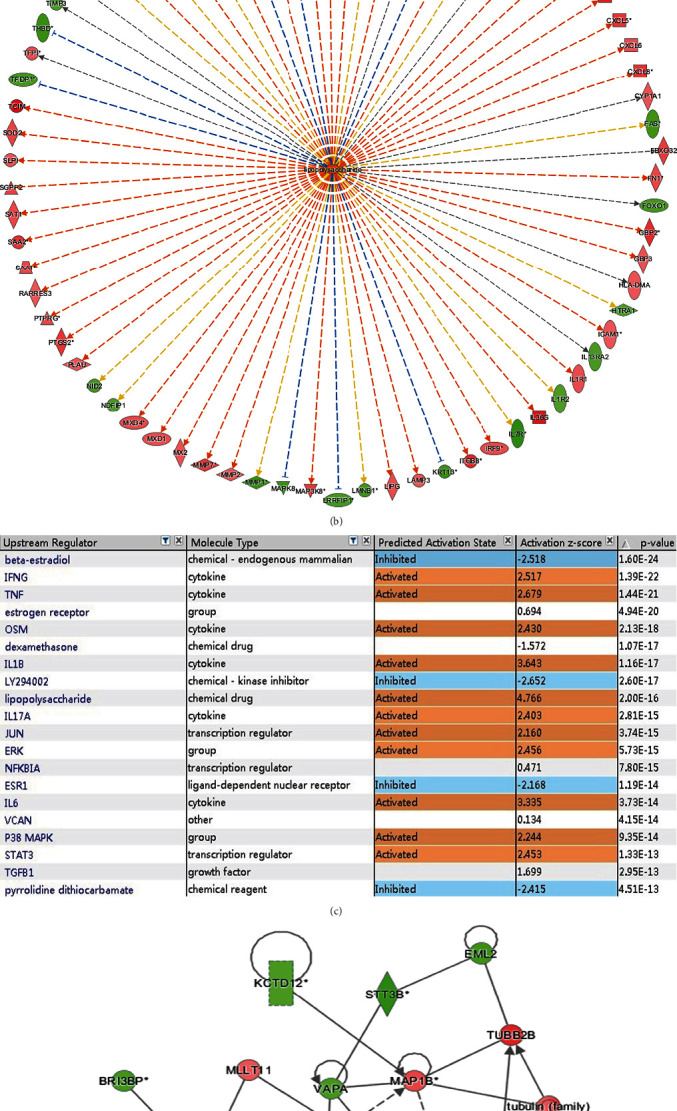
The relative regulatory molecules of *Nuclear protein 1* evaluated by microarray. We used gene chip to explore the expression profile of relative molecules after *Nuclear protein 1* interference. Cluster map panoramic display of differential molecule distribution was indicated in (a). Among them, one of the most up-regulated molecules was lipopolysaccharide (c). Network diagram of lipopolysaccharide was shown in (b). The downstream path network diagram with significant changes was deprived in (d).

We used Affymetrix microarray assays to explore the expression profiles of relative molecules after RNAi silencing of *Nuclear protein 1*. The heat maps of hierarchical clustering of the two groups of samples of KD and normal control (NC) were selected by using the expression profiles of differential genes screened according to the criteria of fold change ≥1.5 and false discovery rate (FDR) <0.05. The cluster panoramic map of differential molecule distribution is shown in Figure 4(a). The tree structure indicated the aggregation of the expression patterns of different genes: red represented that the expression of genes was relatively up-regulated, green represented that the expression of genes was relatively down-regulated, and black indicated no significant change in gene expression. One of the most up-regulated molecules was lipopolysaccharide (Figure 4(c)).  Figure 4(b) shows the network diagram of lipopolysaccharide, and Figure 4(d) shows the downstream path network diagram with significant changes.

### 3.4. Identification of *Nuclear Protein 1*-Related Proteins

**Figure 5:**
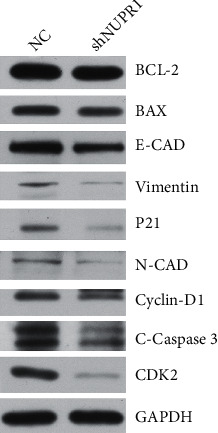
Verification of downstream proteins after *Nuclear protein 1* interference investigated by western blotting. In *Nuclear protein 1* interference group, key proteins in apoptosis (BCL-2), cell cycle (cyclin-D1, CDK2), and epithelial–mesenchymal transformation (vimentin, N-cadherin) were all diminished (*P* < 0.05).

**Figure 6:**
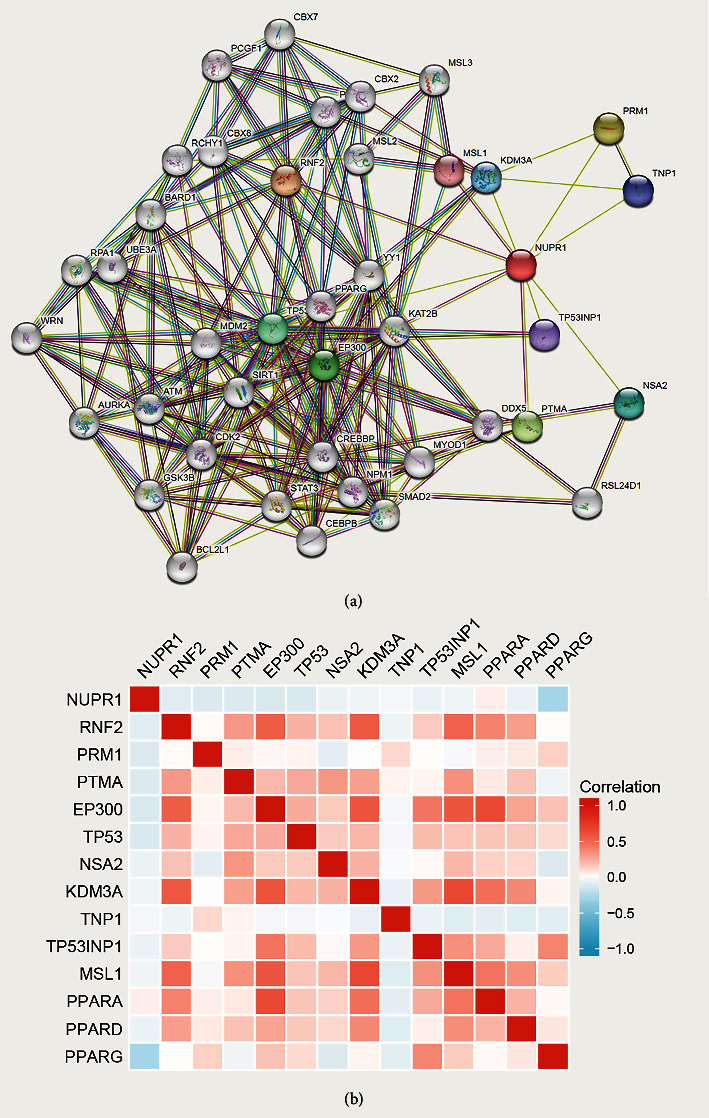
The crosstalk of *Nuclear protein 1* investigated by bioinformatic tools. The STRING database indicated that more than 30 proteins were involved in the correlation with *Nuclear protein 1* (a). PPARG was also the associated gene with *Nuclear protein 1* (b).

We used western blotting to explore the downstream proteins after *Nuclear protein 1* interference. In the *Nuclear protein 1* interference group, key proteins in apoptosis (BCL-2), cell cycle (cyclin-D1, CDK2), and Epithelial–Mesenchymal Transition (EMT) (vimentin, N-cadherin) were all diminished (*P* < 0.05, Figure 5). We applied the STRING database to demonstrate more related proteins associated with *Nuclear protein 1*, indicating that more than 30 proteins were involved in the correlation with *Nuclear protein 1* (Figure 6(a)). In addition, peroxisome proliferator-activated receptor *γ* (PPARG) was found to be associated with *Nuclear protein 1* (Figure 6(b)).

### 3.5. GSEA and Bioinformatic Analyses of *Nuclear Protein 1*-Related Signaling Pathways

**Figure 7:**
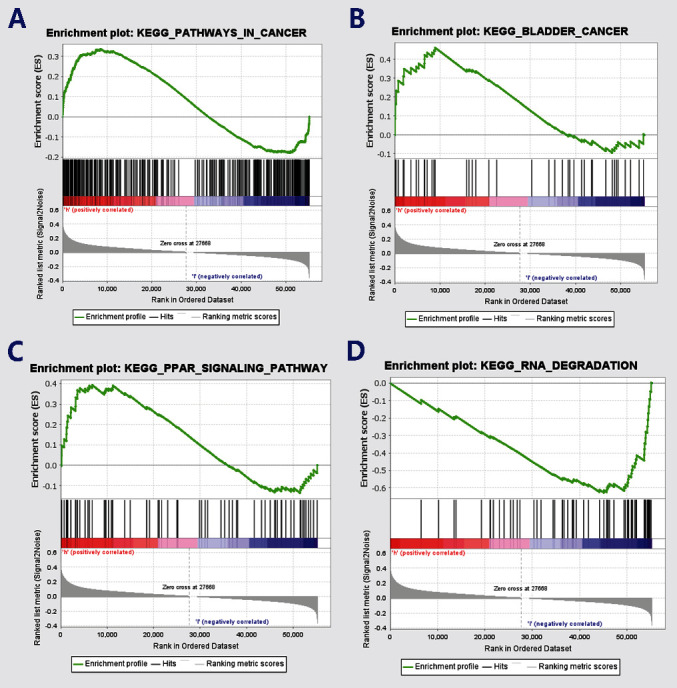
*Nuclear protein 1* is associated with PPAR signaling pathway by Gene Set Enrichment Analysis (GSEA). Results from the GSEA revealed that the signaling pathways in cancer (a) were associated with the expression of *Nuclear protein 1*, especially for BTCC (b). The PPAR (c) signaling pathway and RNA degradation (d) were correlated with a high expression of *Nuclear protein 1*.

**Figure 8:**
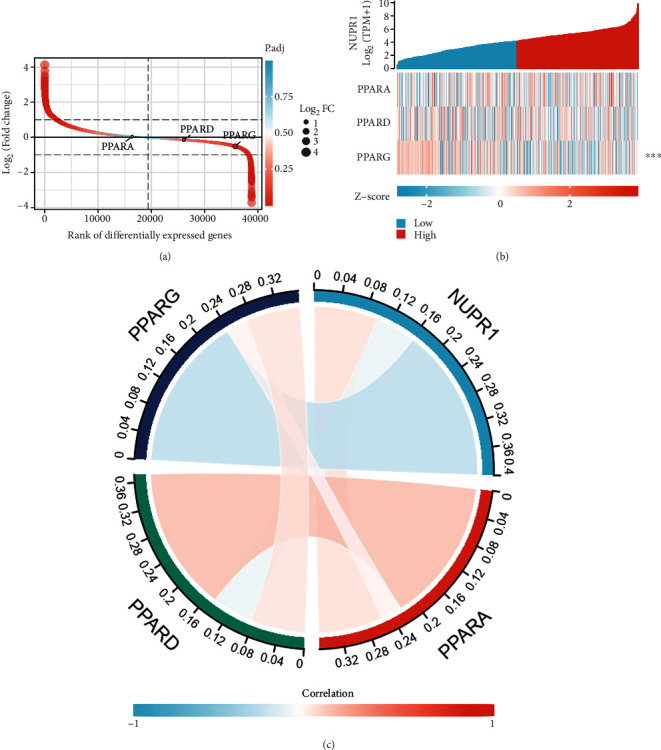
Rank of differentially expressed genes associated with *Nuclear protein 1*. The R language was used to analyze the correlation between *Nuclear protein 1* and PPARA, PPARD, and PPARG (a). *Nuclear protein 1* was significantly correlated with PPARG (b). The correlation chord diagram of *Nuclear protein 1*, PPARG, PPARA, and PPARD was shown in (c).

**Figure 9:**
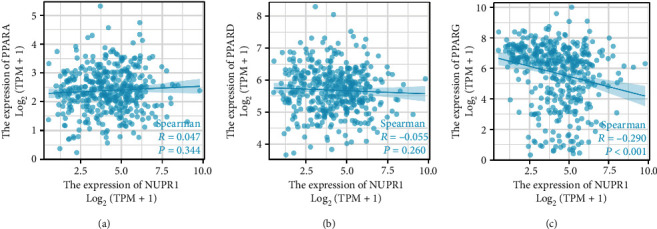
The correlation between *Nuclear protein 1* and PPARA, PPARD, PPARG. Bioinformatic analysis revealed that the expression of *Nuclear protein 1* was negatively correlated with PPARG (*R* = −0.290, *P* < 0.001, (c)), but not with PPARA (*R* = 0.047, *P* = 0.344, (a)) and PPARD (*R* = −0.055, *P* = 0.260, (b)).

**Figure 10:**
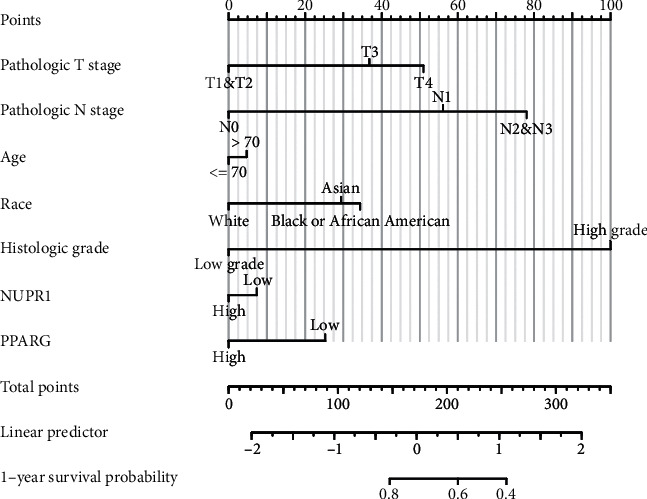
Prognosis nomogram chart of PPARG and *Nuclear protein 1* in BTCC. Prognosis nomogram chart shows that low PPARG expression is an independent risk factor for the prognosis of BTCC.

**Figure 11:**
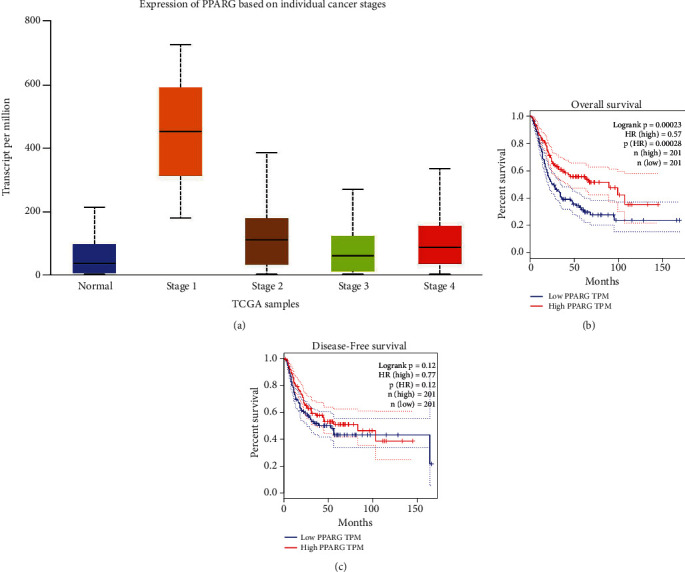
Expression of PPARG in different stage of bladder cancer patients. The expression of PPARG was augmented in patients with early stage bladder cancer (a). Expression of PPARG was diminished in patients with more advanced bladder cancer. Patients with lower PPARG expression had shorter overall survival time than those with higher expression (*P* < 0.05, (b)). No significant difference was revealed for disease-free survival time (*P* > 0.05, (c)).

In order to verify whether the PPAR signaling pathway is associated with *Nuclear protein 1* expression, we employed GSEA to investigate the signaling pathways associated with *Nuclear protein 1*. Results from the GSEA revealed that the signaling pathways in cancer (Figure 7(a)) were associated with the expression of *Nuclear protein 1*, especially for BTCC (Figure 7(b)). The PPAR (Figure 7(c)) signaling pathway and RNA degradation (Figure 7(d)) were associated with high *Nuclear protein 1* expression. We further utilized the R language to explore the differentially expressed genes associated with *Nuclear protein 1*.  Figure 8(a) shows the correlations between *Nuclear protein 1* and PPARA, PPARD, and PPARG. The *Nuclear protein 1* expression was significantly correlated with PPARG in BTCC (Figure 8(b)). The correlation chord diagram of *Nuclear protein 1*, PPARG, PPARA, and PPARD was shown in Figure 8(c). Furthermore, we employed bioinformatic analysis to investigate the association between *Nuclear protein 1* expression and PPAR signaling pathway-related genes, including PPARA, PPARD, and PPARG. The expression of *Nuclear protein 1* was negatively correlated with PPARG (*R* = −0.290, *P* < 0.001, Figure 9(c)), but not with PPARA (*R* = 0.047, *P* = 0.344, Figure 9(a)) and PPARD (*R* = −0.055, *P* = 0.260, Figure 9(b)). Prognosis nomogram chart shows that low PPARG expression is an independent risk factor for the prognosis of BTCC (Figure 10). We further assessed the expression of PPARG in different stage of bladder cancer patients. The expression of PPARG was augmented in patients with early stage bladder cancer (Figure 11(a)). Patients with lower PPARG expression had shorter overall survival time than those with higher expression (*P* < 0.05, Figure 11(b)). No significant difference was revealed for disease-free survival time (*P* > 0.05, Figure 11(c)).

## 4. Discussion

Malignant tumors are still the major diseases that threaten and shorten human life span. Scientists are trying to understand the causes and pathogenesis of cancer, but the results are still not satisfactory [21–24]. Previous retrospective studies have found that different molecular subtypes of BTCC patients show different responses to targeted therapy and different prognoses [25]. The discovery of new genes related to predicting the prognosis of BTCC can provide guidance for the immune microenvironment, lifetime, and chemotherapy responses of patients with BTCC [26]. In this study, we evaluated the expression of *Nuclear protein 1* in the tissues of patients with BTCC from the online database and those enrolled at our center. At the same time, lentivirus-mediated siRNA method was adopted to silence the expression of *Nuclear protein 1* in human BTCC cell lines. We further explored the effect of *Nuclear protein 1* on the biological behavior of BTCC by functional experiments. We anticipate that our findings will offer a new guidance strategy for the early diagnosis and drug therapy of BTCC through investigating the biological function of *Nuclear protein 1* in BTCC.

Biomarkers associated with inflammation and immune activation may help to assess the risk of BTCC. Despite the lack of specificity at present, it would be helpful to predict the routine clinical and pathological prognosis of BTCC in the future. These biomarkers are also expected to improve the outcomes of patients with BTCC. Studies of prognostic models for BTCC patients have shown that many predictive models are promising to improve treatment decisions for BTCC patients. Although many models have not been confirmed in the BTCC patient cohort, some studies have tested the clinical utility of these models and improved the ability to make clinical decisions. The role of inflammation in BTCC has recently been demonstrated, providing insight into the clinical significance of inflammation in preventing the development and progression of BTCC [27–29]. Because BTCC is associated with inflammation, *Nuclear protein 1*, which plays an important role in acute inflammation, may also be associated with the development of BTCC. Hence, it is necessary to investigate the underlying mechanism of these inflammatory factors in malignant tumors to explore the molecular mechanism of drug resistance in cancer cells and provide strategies for the development of effective targets for tumor therapy [30, 31]. The *Nuclear protein 1* participates in numerous malignancy-related processes, including regulation of the cell proliferation, apoptosis, ferroptosis, auto-lysosomal efflux, drug resistance, tumor metastasis, and autophagic-associated cell death [32–38]. Nevertheless, the molecular mechanism of *Nuclear protein 1* in carcinomas has not been clarified. *Nuclear protein 1* dysregulation has been reported in several malignancies, including breast, pancreatic, lung, prostate, and colorectal cancer, as well as glioma [39–44].

To explore the biological function of *Nuclear protein 1* in BTCC, we first used an online database to assess the expression of *Nuclear protein 1*, which was then verified by clinical samples from our centers. We found that *Nuclear protein 1* in high-grade BTCC was augmented in low-grade BTCC. Moreover, *Nuclear protein 1* was positively associated with BTCC stage. Compared with Caucasian patients with BTCC, the *Nuclear protein 1* expression was attenuated in Asian patients. No difference was observed in the expression of *Nuclear protein 1* in patients with different ages. In *Nuclear protein 1* interference group, key proteins in apoptosis (BCL-2), cell cycle (cyclin-D1, CDK2), and epithelial–mesenchymal transformation (vimentin, N-cadherin) were all diminished. Furthermore, we carried out the Affymetrix microarray to explore the relative regulatory molecules and signaling pathways associated with *Nuclear protein 1* in BTCC. Based on the results, lipopolysaccharide was the upstream regulatory factor of *Nuclear protein 1* in BTCC. The results of the present study were in line with those of a previous study conducted by Vasseur et al. [45]. *In vivo* and *in vitro* experiments on the pancreas have confirmed that the mRNA expression of *Nuclear protein 1* can be induced by lipopolysaccharides [46]. Results from *Nuclear protein 1*-knockout in mice showed that *Nuclear protein 1* deficiency hinders normal tissue response to lipopolysaccharides [45]. Prognosis nomogram chart shows that low PPARG expression is an independent risk factor for the prognosis of BTCC. Results from TCGA samples revealed that the expression of PPARG was augmented in patients with early stage bladder cancer and was attenuated in those with more advanced bladder cancer, which is in line with the result from prognosis nomogram chart. Although our previous studies revealed that *Nuclear protein 1* acts as an oncogene in bladder cancer, and the carcinogenic role may be achieved through EMT [47], the crosstalk of *Nuclear protein 1* and PPARG in bladder cancer has not been fully elucidated. Previous literature has shown that PPARG, as a nuclear receptor, is attenuated in basic bladder cancer with muscle invasive, but over-expressed in non-muscle invasive luminal bladder cancer [48]. Additionally, evidence from *in vivo* studies showed the PPARG dependency of bladder urothelial carcinoma and PPARG promotes bladder cancer progression through Sonic Hedgehog signaling-related cellular autonomic mechanisms [49]. In the current study, we found that the PPAR signaling pathway and RNA degradation were correlated with a high expression of *Nuclear protein 1*. Bioinformatic analysis revealed that the expression of *Nuclear protein 1* was negatively correlated with PPARG, but not with PPARA and PPARD. The expression of PPARG was augmented in patients with early stage bladder cancer. Patients with lower PPARG expression had shorter overall survival time than those with higher expression. There are some limitations that need to be mentioned. First, we found that lipopolysaccharide is the upstream regulatory factor of *Nuclear protein 1*; however, the specific regulatory mechanisms of lipopolysaccharides and *Nuclear protein 1* in BTCC tissues were warranted to be further elucidated by more functional experiments. Second, it is reasonable to assess the *Nuclear protein 1* expression in BTCC patients before and after chemotherapy. Nevertheless, due to lack of patients' authorization, we are unable to deal with it at this time. Third, further experiments are needed to confirm the molecular mechanism of *Nuclear protein 1* and PPARG in BTCC in more details.

## 5. Conclusion

The results of the current study indicate that *Nuclear protein 1* is positively associated with the malignancy degree of BTCC. Compared with Caucasian patients with BTCC, the *Nuclear protein 1* expression is attenuated in Asian patients. The Affymetrix microarray showed that lipopolysaccharide is the upstream regulatory factor of *Nuclear protein 1* in BTCC. The expression of *Nuclear protein 1* is associated with PPAR pathways, and *Nuclear protein 1* expression is negatively correlated with PPARG.

## Data Availability

The original data of the study can be acquired from the corresponding authors when they got a rational request.

## Supplementary Materials

Supplementary Materials Supplemental Table 1. Baseline data sheet of enrolled bladder transitional cell carcinoma patients in TCGA database.

Supplementary Materials Supplemental Table 2. Logistic regression model was used to analyze the Odds Ratio (OR) of different characteristics.
