# Supplementary material for: Nuclear Protein 1 Expression Is Associated with PPARG in Bladder Transitional Cell Carcinoma
Source: PPAR Res. 2023 May 8;2023:6797694. doi: 10.1155/2023/6797694 (PMC10185424; doi:10.1155/2023/6797694)
Supplement: Supplementary Materials — Supplemental Table 1. Baseline data sheet of enrolled bladder transitional cell carcinoma patients in TCGA database. [file 6797694.f1.docx]

**Supplemental Table 2. Logistic regression model was used to analyze the Odds Ratio (OR) of different characteristics**

| **Characteristics** | **Total(N)** | **Odds Ratio (OR)** | ***P* value** |
| --- | --- | --- | --- |
| T stage (T3&T4 vs. T1&T2) | 380 | 2.032 (1.316-3.158) | 0.001 |
| N stage (N1&N2&N3 vs. N0) | 370 | 1.782 (1.159-2.756) | 0.009 |
| M stage (M1 vs. M0) | 213 | 2.614 (0.764-10.248) | 0.135 |
| Gender (Male vs. Female) | 412 | 0.883 (0.569-1.368) | 0.577 |
| Race (White vs. Asian) | 374 | 3.600 (1.812-7.685) | <0.001 |
| Age (>70 vs. <=70) | 412 | 1.217 (0.825-1.798) | 0.322 |
| BMI (>25 vs. <=25) | 364 | 1.491 (0.982-2.272) | 0.061 |
| Histologic grade (High Grade vs. Low Grade) | 411 | 6.513 (2.162-28.124) | 0.003 |
| Subtype (Papillary vs. Non-Papillary) | 409 | 0.483 (0.315-0.734) | <0.001 |
| Smoker (Yes vs. No) | 401 | 1.005 (0.647-1.561) | 0.983 |

Each row in the table represents a binary logistic regression model. The independent variable is NUPR1[ENSG00000176046]. The dependent variable corresponds to its Characteristics.
