# Supplementary material for: Nuclear Protein 1 Expression Is Associated with PPARG in Bladder Transitional Cell Carcinoma
Source: PPAR Res. 2023 May 8;2023:6797694. doi: 10.1155/2023/6797694 (PMC10185424; doi:10.1155/2023/6797694)
Supplement: Supplementary Materials — Supplemental Table 2. Logistic regression model was used to analyze the Odds Ratio (OR) of different characteristics. [file 6797694.f2.docx]

**Supplemental Table 1. Baseline data sheet of enrolled bladder transitional cell carcinoma patients in TCGA database.**

| **Characteristics** | **Low expression of *Nuclear protein 1*** | **High expression of *Nuclear protein 1*** | ***P* value** |
| --- | --- | --- | --- |
| n | 206 | 206 |  |
| T stage, n (%) |  |  | < 0.001 |
| T1 | 1 (0.3%) | 4 (1.1%) |  |
| T2 | 77 (20.4%) | 41 (10.8%) |  |
| T3 | 81 (21.4%) | 115 (30.4%) |  |
| T4 | 28 (7.4%) | 31 (8.2%) |  |
| N stage, n (%) |  |  | 0.011 |
| N0 | 132 (35.9%) | 106 (28.8%) |  |
| N1 | 22 (6%) | 24 (6.5%) |  |
| N2 | 27 (7.3%) | 50 (13.6%) |  |
| N3 | 2 (0.5%) | 5 (1.4%) |  |
| M stage, n (%) |  |  | 0.095 |
| M0 | 116 (54.7%) | 85 (40.1%) |  |
| M1 | 3 (1.4%) | 8 (3.8%) |  |
| Age, n (%) |  |  | 0.427 |
| <= 70 | 120 (29.1%) | 112 (27.2%) |  |
| > 70 | 86 (20.9%) | 94 (22.8%) |  |
| Race, n (%) |  |  | < 0.001 |
| Asian | 33 (8.4%) | 11 (2.8%) |  |
| Black or African American | 9 (2.3%) | 14 (3.5%) |  |
| White | 151 (38.2%) | 177 (44.8%) |  |
